# Strengthening resistance to the COVID-19 pandemic and fostering future resilience requires concerted action on obesity

**DOI:** 10.1080/16549716.2020.1804700

**Published:** 2020-08-24

**Authors:** Angela M. Jackson-Morris, Rachel Nugent, Johanna Ralston, Olivia Barata Cavalcanti, John Wilding

**Affiliations:** a Center for Global Noncommunicable Diseases, RTI International, Seattle, WA, USA; b World Obesity Federation, London, UK; c Department of Obesity and Endocrinology, University of Liverpool, Liverpool, UK

**Keywords:** COVID-19, obesity, noncommunicable diseases, resilience, metabolic disease, LMIC, recommendations, global action

## Abstract

Initial observations showed that people with chronic noncommunicable diseases were at heightened risk of severe COVID-19 and adverse outcomes. Subsequently, data from various countries have revealed obesity as an independent and significant factor, with people who are overweight/have obesity significantly more likely to be hospitalized, require ICU treatment, and to die. Notably, this additional risk applies to younger people relative to the general COVID-19 risk profile. This paper sets out the evidence of greater risk of poor COVID outcomes for people who are overweight/have obesity, indication of reduced treatment and support for obesity self-management where it existed prior to COVID-19, and highlights the dearth of specific guidance and measures to mitigate the impacts of COVID-19 upon people with obesity. We identify the health, social and economic impacts that this specific vulnerability creates relative to COVID-19 outcomes. Reduced national and global pandemic resilience due to high obesity prevalence should spur governments and funders to provide urgent specific protection and support for people with overweight/obesity, and to commission rapid research to identify effective prevention and reduction measures. We set out priorities for action on obesity to begin compensating for years of underfunding and inadequate policy attention in the face of escalating obesity across countries of all income groups and world regions.

## Background

Insufficient action to address global obesity over decades is now being ruthlessly exposed by the COVID-19 pandemic. High obesity levels amplify the pressures the pandemic imposes on health systems. There is an urgent need to strengthen obesity prevention and self-management to strengthen longer-term health system resilience and reduce population health impacts, especially among the most vulnerable.

The bigger picture from the pre-COVID-19 landscape clearly demonstrates that the economic, social, and health costs of obesity are growing and are increasingly a development challenge [1,2]. Efforts to build evidence on effective ways to reduce and prevent obesity have been neglected for decades, according obesity lower priority within health plans and budgets. Obesity has often been politically de-prioritized with the issue cast as a failing of individuals rather than a result of social and economic factors and a collective responsibility [3]. Political will to address obesity has further been undermined by the influence of commercial interests, such as ultra-processed food and drink businesses, as well as the challenges of reshaping the built environment to create urban spaces that enbable active living.

We set out the case for governments, researchers, and funders to act to better protect people who have obesity during the COVID-19 pandemic and to lay foundations to reduce this population vulnerability in the years after. We suggest specific priorities for action within pandemic constraints.

Globally, obesity has nearly tripled since 1975. By 2016 almost 2 billion adults were overweight (including 650 million with obesity) [4]. The issue spans all regions and income groups, with nearly two-thirds of people with overweight/obesity living in low- and middle-income countries [5]. The COVID-19 pandemic is also global. Early data emphasized the vulnerability of people living with noncommunicable diseases, such as cardiovascular disease, diabetes, and chronic respiratory disease, as being at substantially greater risk of developing severe COVID-19, being hospitalized, requiring critical care, and dying [6]. Obesity is a risk factor for all those diseases. Most countries therefore include large numbers of people who are vulnerable to the severe impacts of COVID-19. In 2016, 67.5% of US adults were overweight, 63.5% in Egypt, and 32% in China [7]. Prior to the escalation in COVID-19 cases in Latin America, Cuevas et al. warned of the sub-continent’s vulnerability due to high obesity prevalence [8].

## Associations between COVID-19 and obesity

Data from various countries indicate that people with obesity who contract COVID-19 are considerably more likely to become severely ill, require hospitalization and ICU treatment, and die compared to people with normal weight. The most frequently used metric is Body Mass Index (BMI) which has differing thresholds in different world regions. The consistent finding across studies is that patients measured as obese in their country are at far greater risk from COVID-19 than non-obese patients. Chinese studies found that hospitalized people with obesity (BMI≥25) had a three-fold greater risk of severe COVID-19 [9], and patients with obesity in Shenzhen had 2.42 higher odds of severe pneumonia compared to normal weight patients [10]. French patients with obesity were 50% more likely to require ICU admission during March compared to the same period in 2019 [11]; and in the UK (March 2020), 72% of people who developed serious or fatal COVID-19 complications were overweight/had obesity, and severity of obesity was independently associated with higher COVID-19 mortality after correction for other risk factors [12]. US data (March 2020) found that obesity was a factor among almost half (48.3%) of hospitalized COVID-19 patients and was the most prevalent condition among patients aged 18–49 years [6]. Alarmingly, younger patients with obesity have been found to be at high risk of severe COVID-19 [13].

Obesity is an independent risk factor for mortality [14], yet this is often poorly reflected in health system data. The fact that hypertension and smoking were identified very early on as important COVID-19 risk factors, whereas it took months longer to identify the independent role of obesity, may partly reflect the need to disentangle the role of obesity from type 2 diabetes with which it frequently co-exists [15,16]. Less systematic obesity measurement in non-specialist settings and national surveys has also previously been highlighted [16]. Even in high-income countries with electronic health records, non-recording of BMI among COVID cases has impeded a comprehensive picture of the extent and patterning of the association [17].

COVID-19 has exposed fatal weaknesses in the most advanced health systems, leaving little room to address seemingly less urgent problems such as the ongoing burden of ill health due to obesity [18]. Since the start of the pandemic, treatment and support for the chronic NCDs prevalent among people with obesity/overweight, such as diabetes and cardiovascular disease clinics, have receded [19], exacerbating risk of strokes and ‘heart attacks’ and leaving NCD patients without guidance on symptoms and self-management, including weight. Help-seeking from primary care other than for urgent and COVID-19 needs has actively been deterred in many countries, while fear of infection has deterred help-seeking even where services remained open. This first line of support was therefore absent at a time when lockdowns created more challenging circumstances for weight self-management. Specialist weight services and structured patient programs did not exist in many countries prior to the pandemic [20], and existing programs have been halted or reduced [21], leaving a large population without support to manage their condition.

This compounds the pre-existing disadvantage that limits treatment provision and effectiveness for people with obesity, resulting from stigma, provider bias, inconsistent healthcare pathways, unaffordable insurance, and lack of equipment/processes to care effectively for people who have obesity [22]. The current pandemic has highlighted the vulnerability of Black and Asian people and Minority Ethnic groups [23,24], who have experienced the highest COVID-19 mortality. The overlap between higher obesity among people of color and increased vulnerability to COVID-19 complications requires urgent attention. Worse COVID-19 outcomes among men compared to women have also been found [25] and understanding how and why gender and obesity interact will be an important area for research.

Lockdowns and social isolation may exacerbate ‘root’ factors that contribute to obesity, particularly an obesogenic environment in which healthier food and activity patterns are more difficult to achieve [26], and the impact that adverse life events and poor mental wellbeing can have upon weight [27]. Authors from various countries and regions, including Egypt, Latin America, Romania, and the UK have highlighted the danger of high obesity prevalence being exacerbated by the pandemic [8,23,28,29]. The pandemic affects what people are eating and drinking, and reduces physical activity – effects that are likely to be magnified among people on lower incomes, due to more limited food choices as supply chains are disrupted, food price hikes occur, and people have less access to indoor or outdoor space. It has been suggested that misinformation about transmission risk may negatively impact on breastfeeding – an important protective factor against later life obesity [8].

During the pandemic, attention is focused immediately upon reducing transmission, mitigating impact, and finding a treatment or vaccine. Nonetheless, high mortality and hospitalization statistics of people with obesity suggest the need to swiftly develop specific measures for COVID-19 prevention and obesity self-management. These are summarized in Box 1.

**Box 1.** Immediate response recommendations.

Rapid research to understand the contribution of obesity to COVID-19 and to guide establishment of remote self-management support and treatment; Research to understand the additional burden of COVID-19 between different ethnic and socio-economic groups and the role of obesity in this vulnerability; Information and advice for people with obesity on COVID-19 protection, self-care and self-management to limit weight gain and NCD risk; Specific guidance to primary care providers on preventive advice to patients with obesity; guidance to acute care providers on added risk and care needs of patients with obesity; National health communication campaign to advise, encourage and guide weight loss attempts to reduce COVID-19 risks among people with obesity; Service providers (primary care / specialist) to adopt digital service delivery to provide advice and support to patients with obesity; Regulate commercial philanthropy ‘corporate social responsibility’ during the pandemic to prevent promotion of ultra-processed food and drinks and associated brands; Government to prioritize affordable healthier food availability during lockdowns.

## Beyond COVID-19 – reducing vulnerability ‘between pandemics’

Beyond the immediate, COVID-19 should be heard as a warning siren, rousing governments and multilateral agencies to lay foundations for concerted effective measures at scale to reduce and prevent obesity. Until now there has been chronic under-investment in building the evidence on effective ways to reduce and prevent obesity, and obesity has not been accorded sufficient priority within health plans and budgets to catalyse meaningful national level action. Policymakers and ministries of finance have often failed to appreciate that the health, social and economic costs of obesity far outstrip revenue from the manufacture, promotion and sale of unhealthy food and drink and the cost of re-modelling living environments, to promote active living. What has been changed by COVID-19? And how can meaningful action commence while combatting the pandemic?

COVID-19 has shown the significant vulnerability that the increasing unchecked prevalence of NCDs and their risk factors creates to infectious diseases on a scale previously unseen. This has illustrated the unsustainability of high obesity rates in terms of the risk it poses to social, economic and health systems. Foundations for effective action can be laid, even during the pandemic. These are outlined in Box 2. The pandemic offers yet another compelling reason to strengthen efforts to slow the global rise of obesity.

**Box 2.** Laying Foundations for Future Resilience –Recommendations.

Governments to commit to greater priority for obesity in national NCD plans and budgets; Funders to commit finance for research to better understand obesity and to design and test interventions for specific national contexts and population groups; Establish national inter-sectoral taskforces to identify cross-cutting measures to improve access to healthier, affordable nutrition; Governments to commit to restricting promotion / sponsorship of unhealthy products, while supporting commercial research and development to shift production towards healthier products.

## Paper context

Obesity has been found to be an independent factor for adverse COVID-19 outcomes. High obesity prevalence in many countries augments pandemic vulnerability with grave health, social and economic consequences. Yet guidance, treatment and support have been scarce or reduced during COVID-19; and follows years of underinvestment, de-prioritization, and inadequate national action. We identify immediate and forward-looking priorities for action for funders and governments to reduce obesity and strengthen resilience.
